# Annotation of enhanced radiographs for medical image retrieval with deep convolutional neural networks

**DOI:** 10.1371/journal.pone.0206229

**Published:** 2018-11-12

**Authors:** Obioma Pelka, Felix Nensa, Christoph M. Friedrich

**Affiliations:** 1 Department of Computer Science, University of Applied Sciences and Arts Dortmund (FHDO), Dortmund, NRW Germany; 2 Faculty of Medicine, University of Duisburg-Essen, Essen, NRW, Germany; 3 Department of Diagnostic and Interventional Radiology and Neuroradiology, University Hospital Essen, Essen, NRW, Germany; 4 Institute for Medical Informatics, Biometry and Epidemiology (IMIBE), University Hospital Essen, Essen, NRW, Germany; University of Craiova, ROMANIA

## Abstract

The number of images taken per patient scan has rapidly increased due to advances in software, hardware and digital imaging in the medical domain. There is the need for medical image annotation systems that are accurate as manual annotation is impractical, time-consuming and prone to errors. This paper presents modeling approaches performed to automatically classify and annotate radiographs using several classification schemes, which can be further applied for automatic content-based image retrieval (CBIR) and computer-aided diagnosis (CAD). Different image preprocessing and enhancement techniques were applied to augment grayscale radiographs by virtually adding two extra layers. The Image Retrieval in Medical Applications (IRMA) Code, a mono-hierarchical multi-axial code, served as a basis for this work. To extensively evaluate the image enhancement techniques, five classification schemes including the complete IRMA code were adopted. The deep convolutional neural network systems Inception-v3 and Inception-ResNet-v2, and Random Forest models with 1000 trees were trained using extracted Bag-of-Keypoints visual representations. The classification model performances were evaluated using the ImageCLEF 2009 Medical Annotation Task test set. The applied visual enhancement techniques proved to achieve better annotation accuracy in all classification schemes.

## Introduction

With respect to the last decade, ten times more medical images are taken, increasing the number of images per body region per patient to 200–1000 [1]. This huge increase can be traced back to two major facts: rapid advances in technology and significant importance of medical images. Medical images contain relevant information that is valuable to physicians. It provides a reliable source of anatomical and functional information for accurate diagnosis, effective treatment planning as well as research work [2, 3]. The advances of software and hardware in information technology sector and digital imaging in the medical domain have made the acquisition and storage of images in hospitals possible [4].

This large image collection aids medical professionals and improves diagnosis. However, radiologists are challenged by the amount of data. They have to maintain a high interpretation accuracy of radiological images, but also maximize efficiency in terms of the increasing number of images per body region. Computer-based assistance is needed for image interpretation, categorization and annotation [5], as these are beneficial for content-based image retrieval (CBIR) systems and computer-aided diagnosis (CAD) [6].

Deep learning techniques [7] have improved prediction accuracies in object detection [8], speech recognition [9] and in domain application such as medical imaging [10, 11]. Hence, two Deep Convolutional Neural Network (dCNN) systems were adopted for image classification. To compare and evaluate the performance of applied dCNN systems, a traditional classifier was modeled in addition.

This paper evaluates the effect of several image enhancement techniques on the prediction accuracy rate on radiographs. To analyze this value, several classification schemes were acquired from the ImageCLEF 2009 Medical Annotation Task dataset. All images used at the training and testing stages were preprocessed with the various presented image enhancement techniques. Finally, the obtained image annotation performance accuracies are compared and discussed.

### Related work

Several approaches to Information Retrieval (IR) in Medical Domain as objective have been designed. KHRESMOI was a large EU-funded project aimed at creating a multilingual and multimodal-based search system for biomedical information and documentation [12]. The GNU Image-Finding Tool (GIFT), an outcome of the Viper Project, enables users to perform query-by-example (QBE) search and improves result quality with relevance feedback [13]. In [14], Parallel Distributed Image Search Engine (ParaDISE) was proposed. This search engine enables the indexing and retrieving of images using present visual and text features. The Lucene Image Retrieval (LIRE), a lightweight open source library, provides image retrieval using visual features such as color and texture [15]. The IRMA-code, a mono-hierarchical multi-axial classification code for medical image was proposed in the Image Retrieval in Medical Applications (IRMA) [16]. The IRMA-code describes the modality of the images, orientation of the image, examined body region and the biological system investigated.

Positive results have been achieved by image preprocessing using input color enhancement techniques. In [17], superior values were obtained by using dual deep convolutional neural networks and color input enhancement [18] to detect malignancy in digital mammography images. As computer-aided assistance is needed in image interpretation [19] and improved prediction accuracies have been obtained using deep convolutional neural networks [7], the objective of this paper is to create an automatic image annotation system using deep learning and image enhancement techniques. These annotated radiographs are fundamental for medical image retrieval systems.

The aim of this presented approach is to apply several image enhancement techniques on radiographs, to increase the overall prediction accuracy of classification models. This is fundamental for implementing image retrieval systems.

## Material

### Dataset

**Fig 1:**
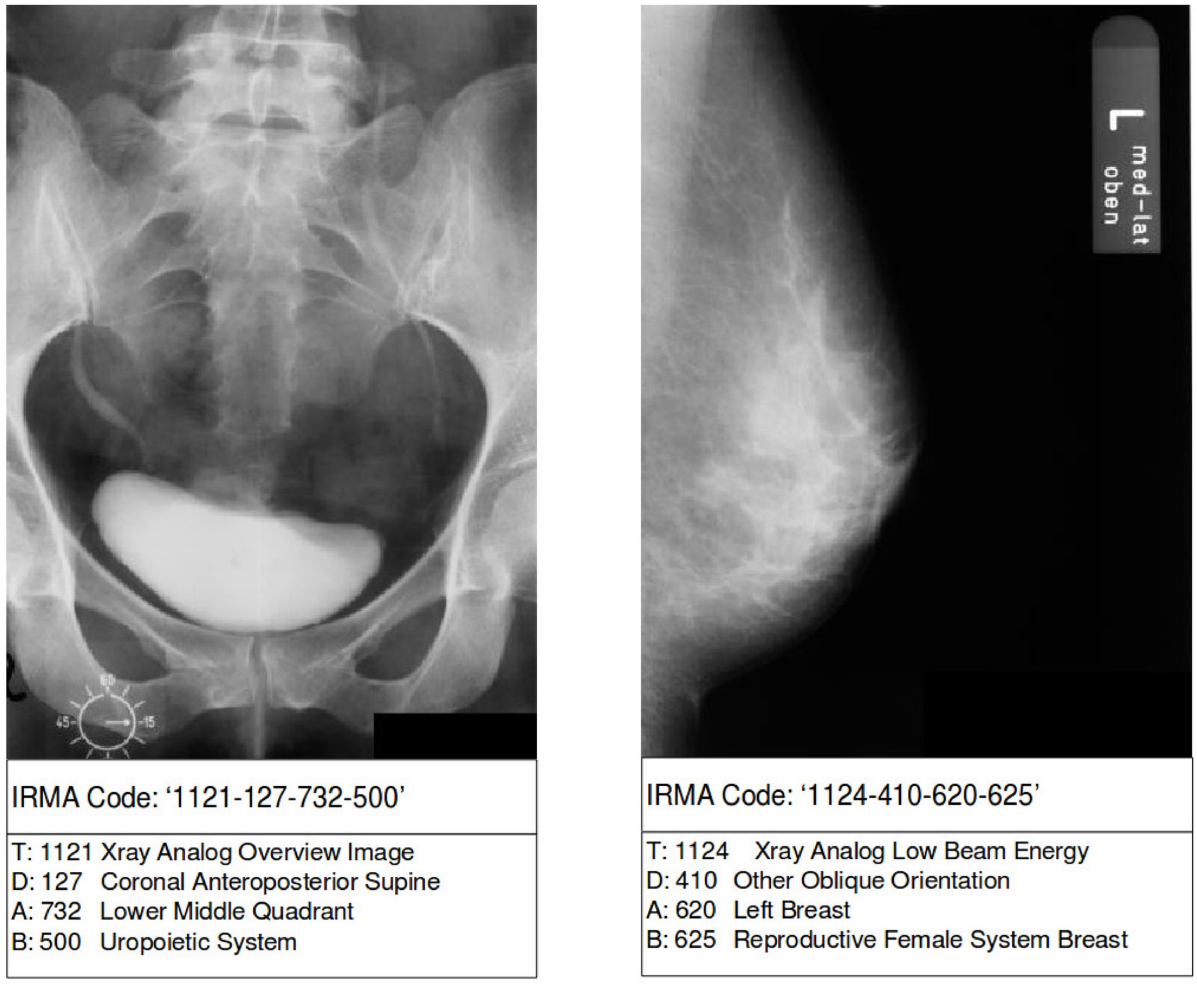
Example of two grayscale radiographs annotated with the 13-digit classification code. Both images were randomly chosen from the ImageCLEF 2009 Medical Annotation Task Training Set. Republished from [21] under a CC BY license, with permission from [RWTH Aachen], original copyright [2009].

The dataset adopted for evaluation was distributed at the ImageCLEF 2009 Medical Annotation task [20, 21]. The training set consists of 12,671 grayscale images and the official evaluation set has 1,732 grayscale images. Each radiograph in the training set is annotated with a 13-character string. Fig 1 shows two radiographs with the annotations *1121-127-732-500* and *1121-410-620-625*, representing “Xray Analog Overview Image; Coronal Anteroposterior Supine; Lower Middle Quadrant; Uropoietic System” and “Xray Analog Low Beam Energy; Other Oblique Orientation; Left Breast; Reproductive Female System Breast”.

### Classification schemes

5 different classification schemes are used for evaluation, which were derived by using the complete IRMA code, as well as splitting the code to its’ four axes.

#### IRMA

The 13-digit code used for annotation is known as the IRMA code and was proposed in [16]. The IRMA coding system is hierarchical and consists of four axes: the technical code (T) for image modality, the directional code (D) for body orientations, the anatomical code (A) referring to body region examined, and the biological code (B) for the biological system examined [16]. The code results in a string of 13 characters, ie. TTTT-DDD-AAA-BBB, which can be seen in Fig 1. The IRMA classification scheme contains altogether 197 individual classes, which represent the total distinct combinations of all four axes.

#### (T) technical scheme

**Fig 2:**
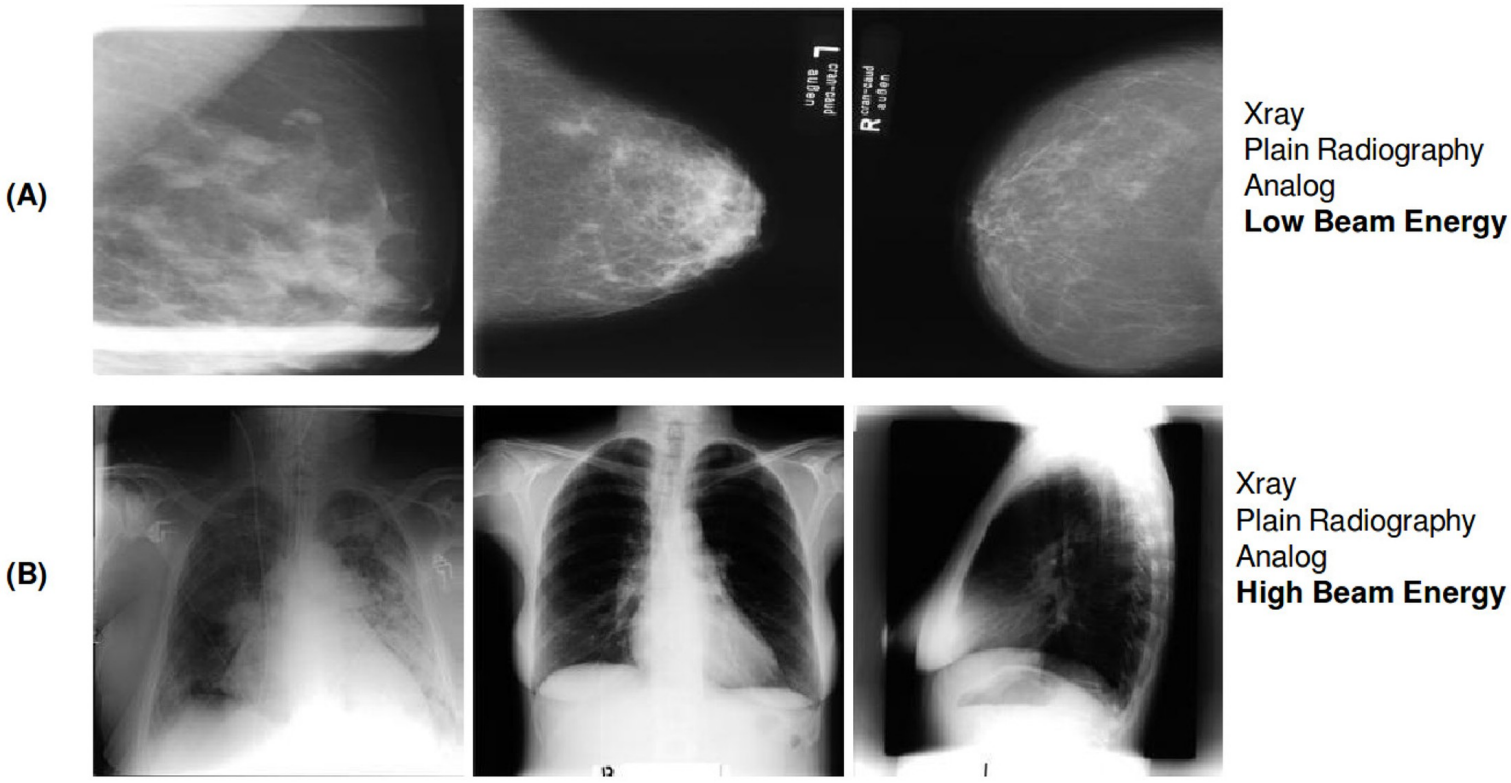
Examples of radiographs annotated with two classes from the T-scheme. (A) shows three images belonging to class ‘1124’ representing ‘Xray; Plain Radiology; Analog; Low Beam Energy’ and (B) displays three images belonging to class ‘1123’ representing ‘Xray; Plain Radiology; Analog; High Beam Energy’. All radiographs were randomly chosen from the ImageCLEF 2009 Medical Annotation Task Training Set. Republished from [21] under a CC BY license, with permission from [RWTH Aachen], original copyright [2009].

The (T) technical classification scheme is the technical axis of the IRMA code. It consists of a 4-character string and denotes physical source, modality position, techniques and sub-techniques [16]. The T-scheme has 6 classes. A random excerpt of radiographs from the training set annotated with the t-scheme is shown in Fig 2.

#### (D) directional scheme

**Fig 3:**
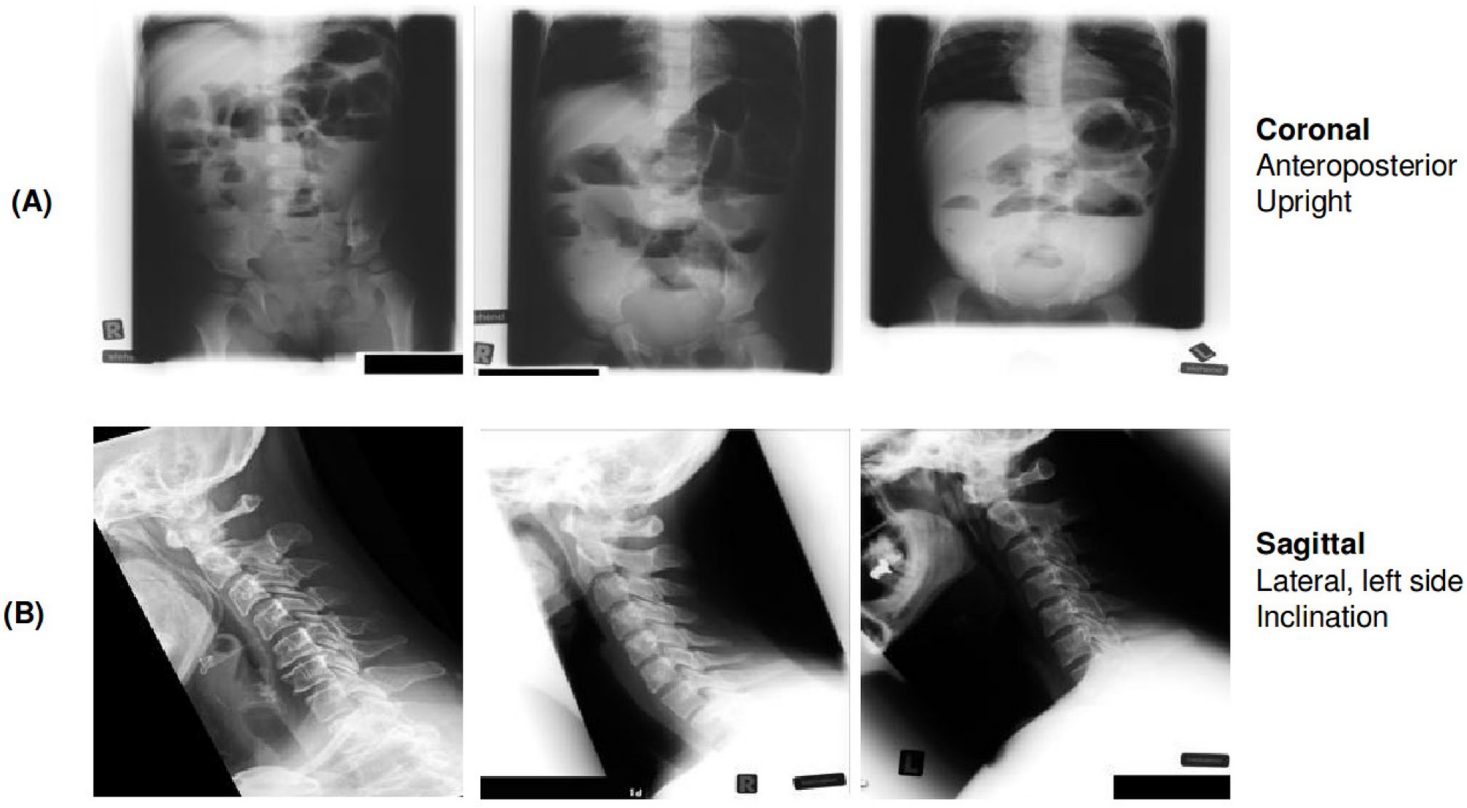
Examples of radiographs annotated with two classes from the D-scheme. (A) shows three image belonging to class ‘125’ representing ‘Coronal; Anteroposterior; Upright’ and (B) displays three images belonging to class ‘228’ representing ‘Sagital; Lateral, left-right; Inclination’. All radiographs were randomly chosen from the ImageCLEF 2009 Medical Annotation Task Training Set. Republished from [21] under a CC BY license, with permission from [RWTH Aachen], original copyright [2009].

The (D) directional classification scheme is a 3-character string and denotes the orientation plane of the radiographs, such as coronal, sagittal and transversal [16]. This scheme is made up of 34 classes. A random excerpt of radiographs from the training set annotated with the d-scheme is shown in Fig 3.

#### (A) anatomical scheme

**Fig 4:**
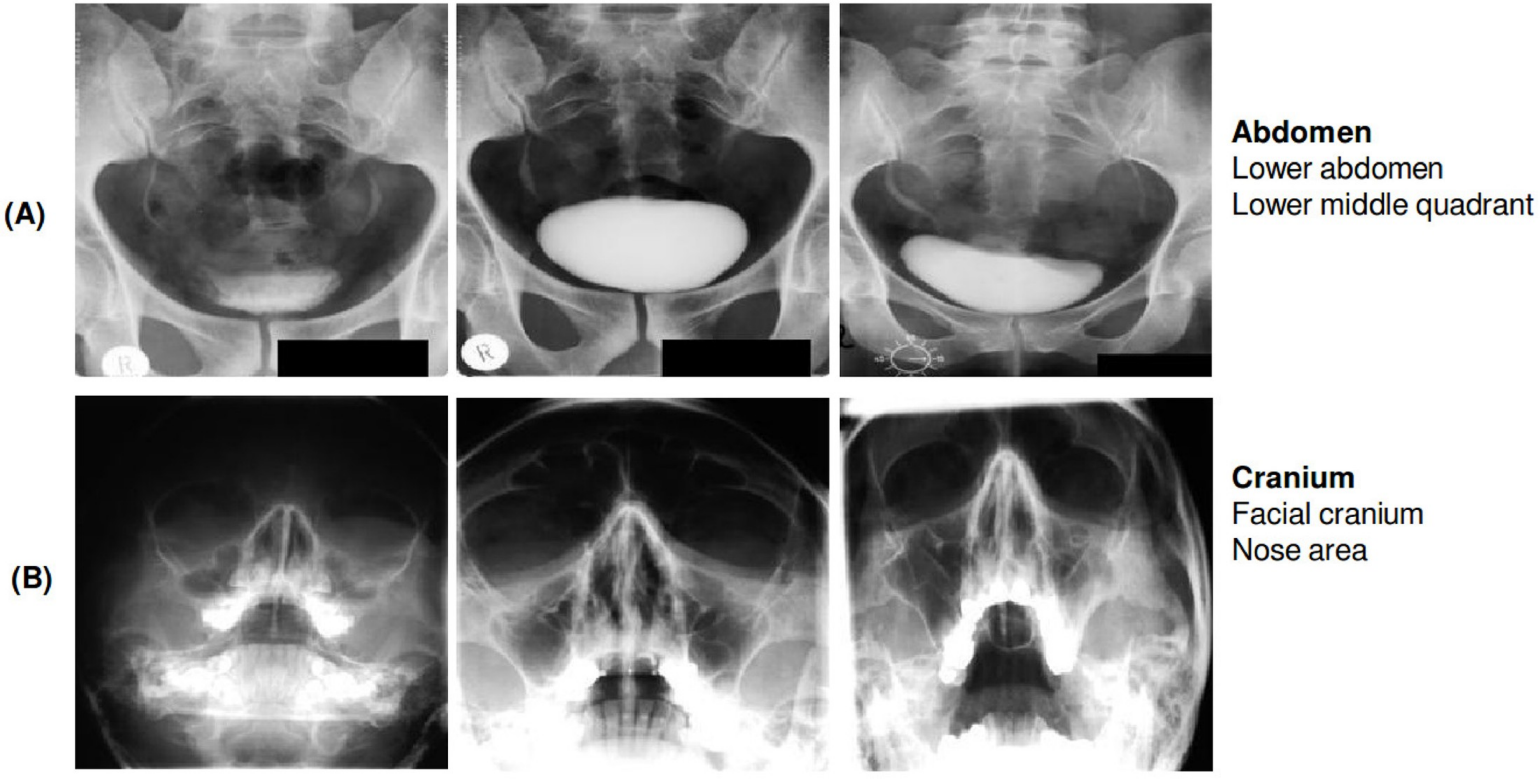
Examples of radiographs annotated with two classes from the A-scheme. (A) shows three images each belonging to class ‘732’ representing ‘Abdomen; Lower abdomen; Lower middle quadrant’ and (B) displays three images belonging to ‘213’ representing ‘Cranium; Facial cranium; Nose area’. All radiographs were randomly chosen from the ImageCLEF 2009 Medical Annotation Task Training Set. Republished from [21] under a CC BY license, with permission from [RWTH Aachen], original copyright [2009].

The (A) classification scheme stands for the complete coding of anatomical regions which are present in the human body. The A-scheme defines nine major body regions, where each region has 2 hierarchical sub-regions [16]. In total, the anatomical scheme has 97 individual classes and each class is represented by a 3-character string. A random excerpt of radiographs from the training set annotated with the a-scheme is shown in Fig 4.

#### (B) biological scheme

**Fig 5:**
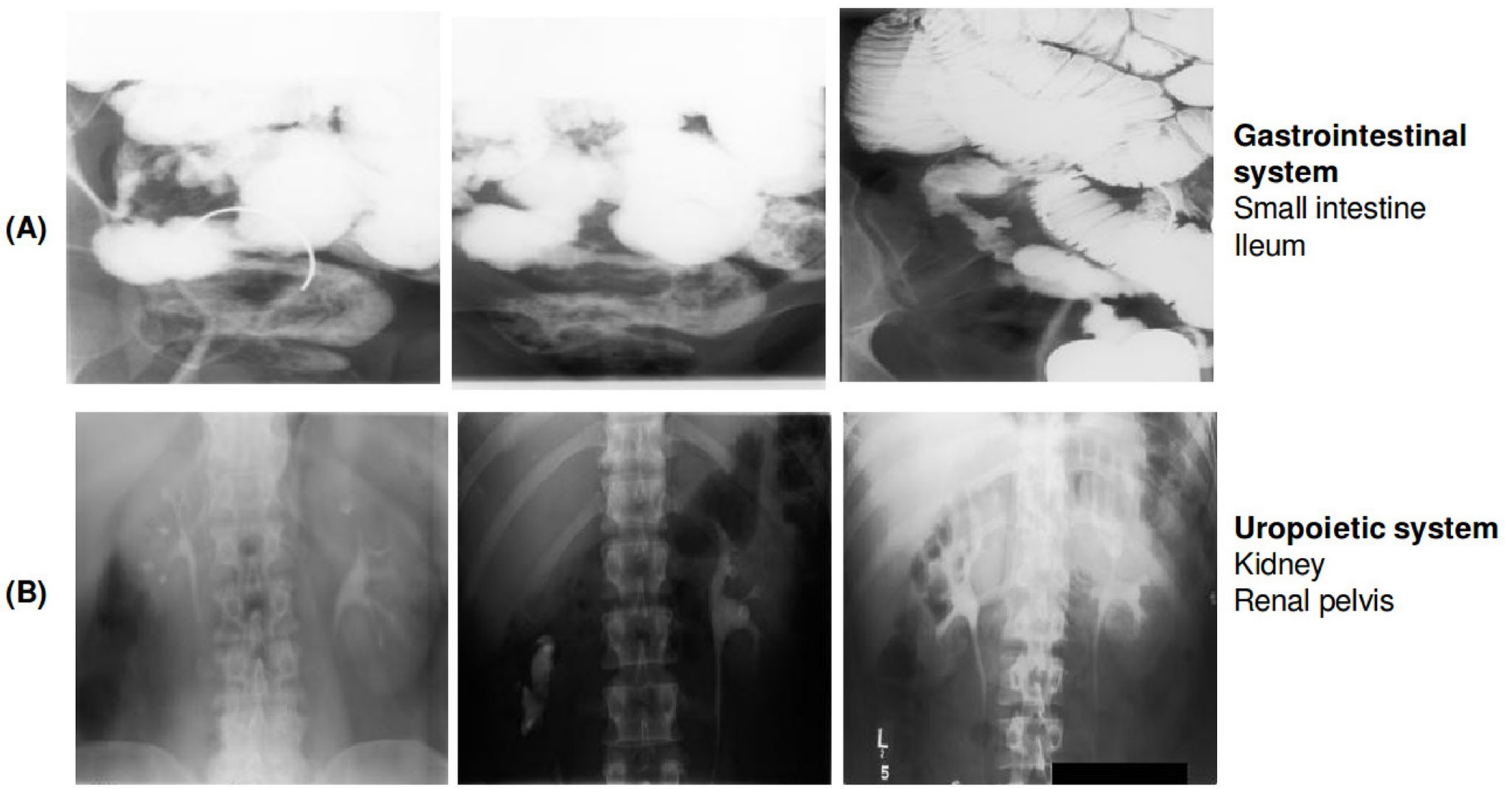
Examples of radiographs annotated with two classes from the B-scheme. (A) shows three imagse belonging to class ‘443’ representing ‘Gastrointestinal system; Small intestine; Ileum’ and (B) displays three images belonging to class ‘512’ representing ‘Uropoietic system; Kidney; Renal pelvis’. All radiographs were randomly chosen from the ImageCLEF 2009 Medical Annotation Task Training Set. Republished from [21] under a CC BY license, with permission from [RWTH Aachen], original copyright [2009].

The (B) biological classification code categorizes the organic system scanned into ten major parts [16]. The B-scheme contains 11 classes and is represented by a 3-character string. A random excerpt of radiographs from the training set annotated with the b-scheme is shown in Fig 5.

## Image enhancement

In this section, the three experiments adopted for enhancing visual representation before the classification and annotation of the radiographs are explained.

### Image layering

For image recognition tasks, convolutional neural networks trained on large datasets produce favorable results. Considering the number of images in the ImageCLEF 2009 Medical Annotation Task, the adaptation of Transfer Learning with pre-trained neural, such as Inception-v3 [22] and Inception-ResNet-v2 [23], networks was chosen. These pre-trained Deep Convolutional Neural Network (dCNN) models were designed to extract amongst other features, color information from the images [24, 25]. However, the radiographs distributed for at the ImageCLEF 2009 Medical Annotation Task are grayscale images and have single color channel with values [0, 255]. To fully utilize the capabilities of dCNNs, two extra color layers are augmented to each radiograph, completing the RGB frames with the enhanced slices.

**Fig 6:**
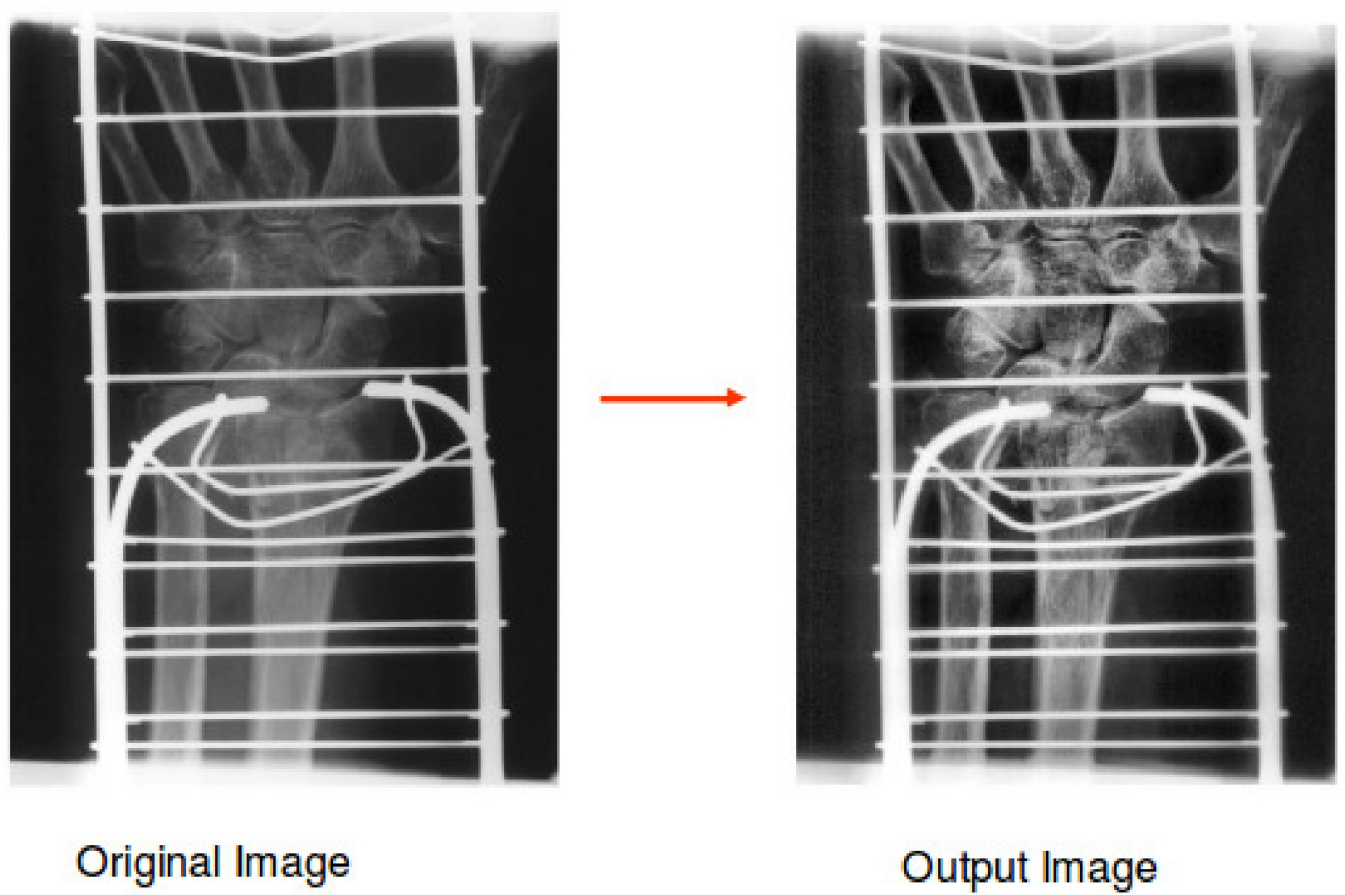
Medical image before and after Contrast Limited Adaptive Histogram Equation (CLAHE) was performed. The radiograph was randomly chosen from the ImageCLEF 2009 Medical Annotation Task Training Set. Republished from [21] under a CC BY license, with permission from[RWTH Aachen], original copyright [2009].

The first extra layer was obtained using the image processing technique: Contrast Limited Adaptive Historization Equation (CLAHE) [18]. CLAHE is a contrast enhancement method, modified from the Adaptive Histogram Equation (AHE). It is designed to be broadly applicable and having demonstrated effectiveness, especially for medical images [26]. Fig 6 displays the original radiograph and the corresponding output image after CLAHE was performed. The CLAHE output images were obtained using the following parameters:

- Number of tiles: [8, 8]
- Contrast enhancement limit: 0.01
- Number of histogram bins: 256
- Range of output data: Full
- Desired histogram shape: Uniform
- Distribution parameter: 0.4

**Fig 7:**
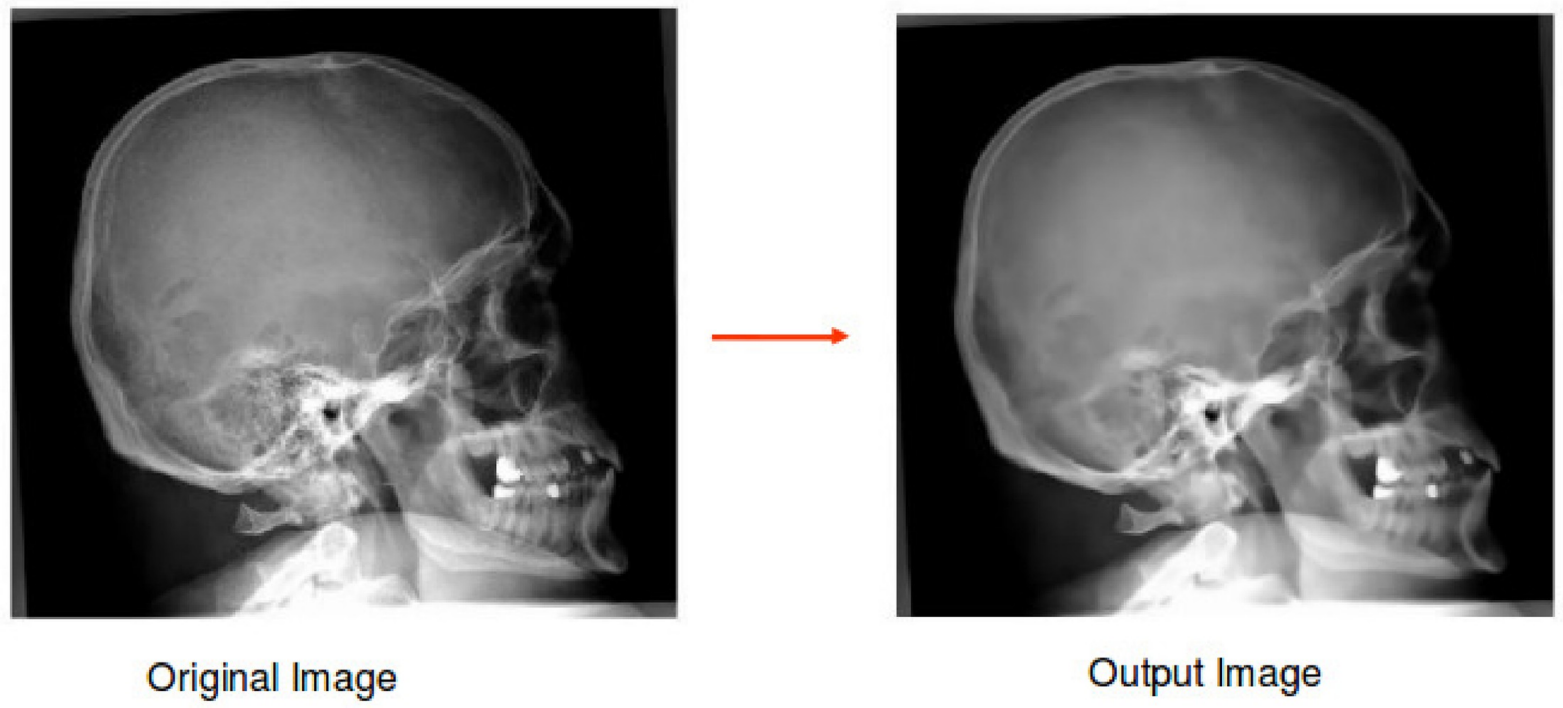
Medical image before and after applying the Non Local Means (NL-MEANS) preprocessing method. The radiograph was randomly chosen from the ImageCLEF 2009 Medical Annotation Task Training Set. Republished from [21] under a CC BY license, with permission from [RWTH Aachen], original copyright [2009].

The second layer was generated by applying the Non Local Means (NL-MEANS) preprocessing method. This is a digital image denoising method, based on a non local averaging of all present pixels in an image [27]. The effect of applying NL-MEANS to a randomly chosen radiograph from the ImageCLEF 2009 Medical Annotation Task Training Set is shown in Fig 7. The NL-MEANS output images were obtained using the following parameters:

- Kernel ratio: 4
- Window ratio: 4
- Filter strength: 0.05

**Fig 8:**
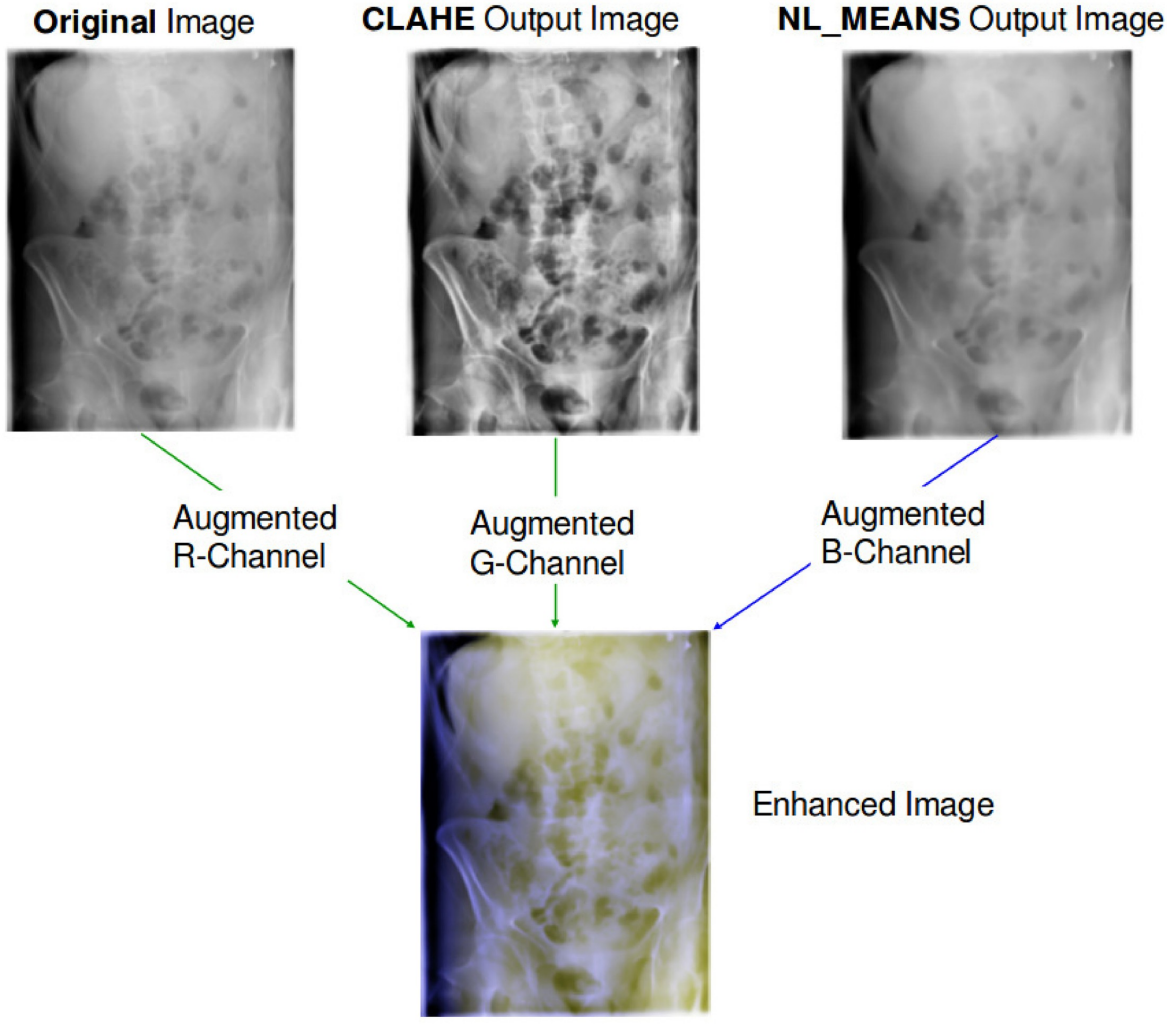
Enhanced grayscale radiograph, by augmenting 2 extra color layers to obtain a RGB-channeled medical image. The radiographs were randomly chosen from the ImageCLEF 2009 Medical Annotation Task Training Set. Republished from [21] under a CC BY license, with permission from [RWTH Aachen], original copyright [2009].

The augmented RGB-Image is obtained by adding the two layers to the original grayscale radiograph, as shown in Fig 8.

### Image padding

**Fig 9:**
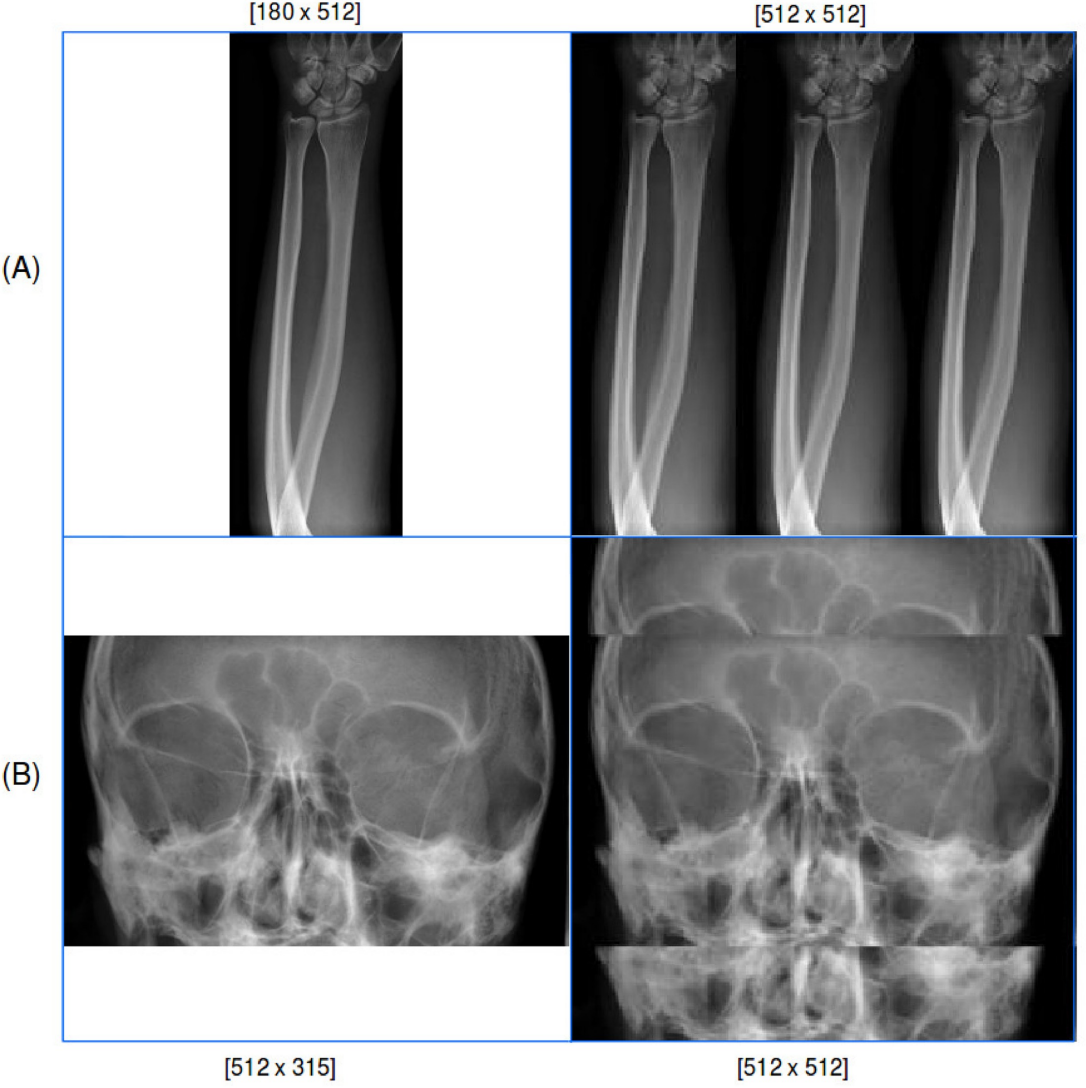
Resized radiographs by padding input images to the defined width and height size [512 x 512]. (A) shows horizontal and (B) vertical padding. The radiographs were randomly chosen from the ImageCLEF 2009 Medical Annotation Task Training Set. Modified from [21] under a CC BY license, with permission from [RWTH Aachen], original copyright [2009].

There are variations regarding the height and width of the radiographs distributed for the ImageCLEF 2009 Medical Annotation Task. The upper and lower extremities are usually narrow with less width size, while head scans are wider with less height size. To obtain size similarity over all images, a fixed size was defined. All radiographs in the dataset were resized to [512 x 512] by padding the input images, which can be seen in Fig 9. The images are padded with their repetition, other alternatives are padding with a constant value or noise as well as image squashing.

**Fig 10:**
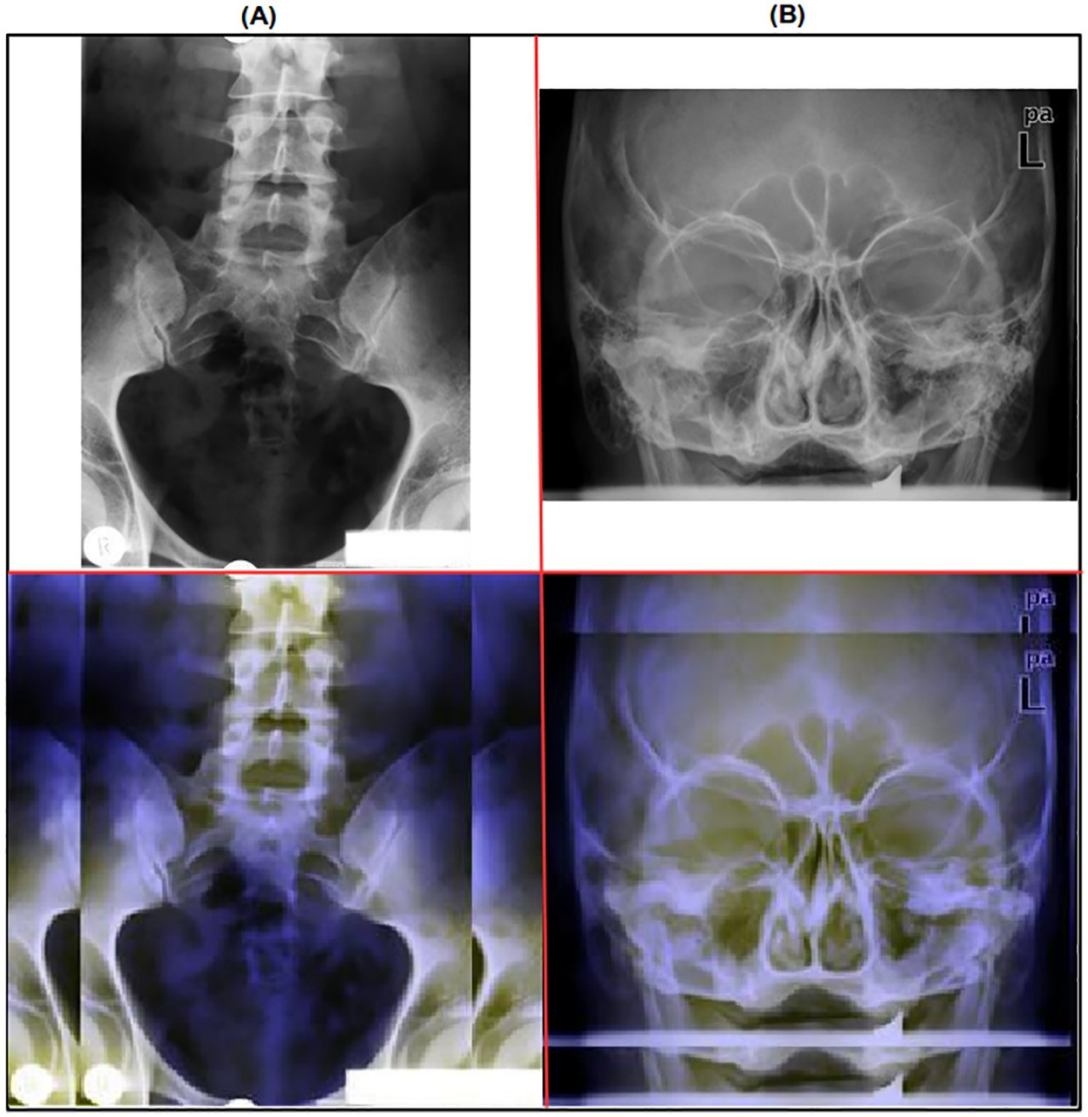
Output image after successively applying the image padding and image layering enhancement techniques. The radiographs were randomly chosen from the ImageCLEF 2009 Medical Annotation Task Training Set. Modified from [21] under a CC BY license, with permission from [RWTH Aachen], original copyright [2009].

Both image layering and padding, as explained in subsections **Image Layering** and **Image Padding**, are applied successively; the output image is shown in Fig 10.

## Classification

### TensorFlow

For the dCNNs, TensorFlow-Slim (TF-slim), a lightweight package for defining, training and evaluating models in TensorFlow [28] with pre-trained models, was adopted. To optimize prediction performance, the models were fine-tuned with all trainable weights and best hyper-parameter configuration in the second training phase.

#### Inception-v3

The pre-trained model Inception-v3 [22] which was trained for the ImageNet [24] Large Visual Recognition Challenge 2012 [29], was used to fine-tune the classification model. To optimize classification accuracy, a grid search was used to obtain best hyper-parameters configurations. For the Inception-v3 classification models, the following hyper-parameter configuration was applied:

- Optimizer: Root Mean Square Propagation (rmsprop)
- Number of epochs: [1. Trainingphase = 2.5; 2. Trainingphase = 25]
- Number of steps: [1. Trainingphase = 1,000; 2. Trainingphase = 10,000]
- Batch size: [1. Trainingphase = 2.5; 2. Trainingphase = 25]
- Learning rate: 0.01
- Learning rate decay type: [1. Trainingphase = fixed; 2. Trainingphase = exponential]
- Weight decay: 0.00004
- Model name: Inception-v3

For all other parameters not mentioned above, the default values as proposed in TF-Slim [28] were adopted.

#### Inception-ResNet-v2

The pre-trained model Inception-ResNet-v2 [23] which is a variation of the Inception-v3 using the ideas presented in [30, 31], was used to fine-tune the classification model. For the Inception-ResNet-v2 classification models, the following hyper-parameter configuration was applied:

- Optimizer: Root Mean Square Propagation (rmsprop)
- Number of epochs: [1. Trainingphase = 2.5; 2. Trainingphase = 25]
- Number of steps: [1. Trainingphase = 1,000; 2. Trainingphase = 10,000]
- Batch size: 32
- Learning rate: 0.01
- Learning rate decay type: [1. Trainingphase = fixed; 2. Trainingphase = exponential]
- Weight decay: 0.00004
- Model name: Inception-ResNet-v2

For all other parameters not mentioned above, the default values as proposed in TF-Slim [28] were adopted.

### Random Forest

Random forest (RF) [32] models with 1000 deep trees were trained to compare accuracy performances amongst classification models. These RF-models were trained using visual image representation obtained with the Bag-of-Keypoints (BoK) [33] approach. For whole-image classification tasks, BoK approach has achieved high classification accuracy results [34, 35]. BoK is based on vector quantization of affine invariant descriptors of image patches [33]. The simplicity and invariance to affine transformation are advantages that come with this approach.

All functions applied to render visual models are from the *VLFEAT* library [36]. Dense SIFT (dSIFT) [37] applied at several resolutions were uniformly extracted with an interval of 4 pixels using the *VL-PHOW* function. Computational time was sped up by computing *k*-means clustering with Approximated Nearest Neighbor (ANN) [38] on randomly chosen descriptors using the *VL-KMEANS* function. This partitions the observations into *k* clusters so that the within-cluster sum of square is minimized.

A maximum number of 20 iterations was defined to allow the *k*-means algorithm to converge and cluster centers were initialized using random data points [39]. A codebook containing 1,000 keypoints was generated as *k* = 1,000. Using the *VL-KDTREEBUILD* function, the codebook was further optimized by adapting a kd-tree with metric distance *L*2 for quick nearest neighbor lookup. Parameters used to tune BoK and RF are:

- Codebook size: 1,000
- Number of descriptors extracted: 1,000
- Visual representation size: 4,000 (2x2 grid)
- Feature size reduction: 4000 to 100 (Principal Component Analysis)
- Number of trees (RF): 1,000
- Ensemble method (RF): Bag

## Results

Image class prediction was computed using five classification schemes: the complete IRMA code and its 4 axes separately. The performance of modeled classifiers on different classification schemes are listed in Tables 1–3, for Random Forest, Inception-v3 and Inception-ResNet-v2, respectively.

**Table 1:** Prediction performance of the Random Forest image classification model on the various image input types. The highlighted accuracies are the best per classification scheme. Evaluation was calculated on the ImageCLEF 2009 Medical Annotation Task Test Set.

Input Image	T-Code	D-Code	A-Code	B-Code	IRMA
Image Padding	97.98%	**63.57%**	53.35%	92.21%	48.67%
Image Padding/Layered	97.52%	62.47%	51.15%	**92.32%**	47.98%
Image Layered	**98.09%**	63.05%	**54.39%**	91.40%	**48.96%**
CLAHE Image	97.69%	60.68%	50.46%	91.97%	45.84%
NLMEANS Image	97.58%	61.89%	49.42%	91.22%	45.09%
Original Image	97.00%	61.64%	51.15%	90.76%	47.11%

**Table 2:** Prediction performance of the Inception-v3 image classification model on the various image input types. The highlighted accuracies are the best per classification scheme. Evaluation was calculated on the ImageCLEF 2009 Medical Annotation Task Test Set.

Input Image	T-Code	D-Code	A-Code	B-Code	IRMA
Image Padding	**99.21%**	76.61%	**60.33%**	**96.39%**	46.39%
Image Padding/Layered	99.06%	**79.11%**	56.67%	95.78%	**47.00%**
Image Layered	98.83%	74.33%	50.22%	93.78%	39.77%
CLAHE Image	98.61%	71.72%	45.77%	93.61%	39.44%
NLMEANS Image	96.78%	69.33%	43.06%	95.22%	40.44%
Original Image	97.89%	70.00%	44.33%	91.50%	39.33%

**Table 3:** Prediction performance of the Inception-ResNet-v2 image classification model on the various image input types. The highlighted accuracies are the best per classification scheme. Evaluation was calculated on the ImageCLEF 2009 Medical Annotation Task Test Set.

Input Image	T-Code	D-Code	A-Code	B-Code	IRMA
Image Padding	99.22%	**78.50%**	57.89	**96.78%**	**51.22%**
Image Padding/Layered	**99.28%**	75.72%	**59.83%**	89.22%	49.83%
Image Layered	98.67%	77.61%	53.44%	92.83%	49.31%
CLAHE Image	98.06%	76.11%	51.22%	95.56%	43.33%
NLMEANS Image	97.00%	70.89%	49.44%	94.61%	42.00%
Original Image	97.33%	73.88%	49.94%	94.67%	42.67%

Evaluation was performed on the official test set and all models were trained with the complete training set distributed at the ImageCLEF 2009 Medical Annotation Task.

The best prediction performances per classifier model and image input obtained on the different classification schemes are displayed in Tables 4–8 for easier understanding. Evaluation was calculated for using the ImageCLEF 2009 Medical Annotation Task test set.

**Table 4:** Best prediction performances for the applied classification models. The classification scheme is (T) technical axis and contains 6 classes.

Input Image	Classifier	Performance
Image Layered	Random Forest	98.09%
Image Padding	Inception-v3	99.21%
Image Layered/Padding	Inception-ResNet-v2	**99.28%**

**Table 5:** Best prediction performances for the applied classification models. The classification scheme is (D) directional axis and contains 34 classes.

Input Image	Classifier	Performance
Image Padding	Random Forest	63.57%
Image Layered/Padding	Inception-v3	**79.11%**
Image Padding	Inception-ResNet-v2	78.50%

**Table 6:** Best prediction performances for the applied classification models. The classification scheme is (A) anatomical axis and contains 97 classes.

Input Image	Classifier	Performance
Image Layered	Random Forest	54.39%
Image Padding	Inception-v3	**60.33%**
Image Layered/Padding	Inception-ResNet-v2	59.83%

**Table 7:** Best prediction performances for the applied classification models. The classification scheme is (B) biological system axis and contains 11 classes.

Input Image	Classifier	Performance
Image Layered/Padding	Random Forest	92.32%
Image Padding	Inception-v3	96.39%
Image Padding	Inception-ResNet-v2	**96.78%**

**Table 8:** Best prediction performances for the applied classification models. The classification scheme is the complete IRMA code, which has 193 classes.

Input Image	Classifier	Performance
Image Layered	Random Forest	48.96%
Image Padding	Inception-v3	47.00%
Image Padding	Inception-ResNet-v2	**51.22%**

## Discussion

It can be seen from all result tables, better prediction accuracies are obtained with the enhanced radiographs. This is observed for all three classification models and all five schemes adopted. However, there is not one enhancement technique that outperforms the rest, it varies with the classification scheme used, which can be explained by the no free lunch theorem [40].

Certain enhancement techniques perform better at some classification schemes. Image Layered achieves best results when trained with the Bag-of-Keywords and Random Forest. Image Padding performs best with models trained with the deep learning system Inception-v3. For models trained with Inception-ResNet-v2, Image Layered/Padding leads to better results. Best prediction performance was obtained with the following model and enhancement technique combination:

- (T) technical: Image Padding and Layering with Inception-ResNet-v2
- (D) directional: Image Padding and Layering with Inception-v3
- (A) anatomical: Image Padding with Inception-v3
- (B) biological: Image Padding with Inception-ResNet-v2
- IRMA: Image Padding with Inception-ResNet-v2

As the number of classes increase, the prediction accuracy rate decreases. The anatomical and IRMA schemes are class imbalanced, having less or no image representing some classes. Hence, the uncertainty of the models is high at these images. The prediction results of the IRMA scheme is lowest, as it contains the highest number of classes of sparse representations. However, a hierarchical classification can be used to tackle this task, as the results in the individual axes perform well.

Following the shown results, a more robust and certain model can be obtained with a balanced class distribution of the images in the training set. An ensemble of models trained with several image enhancement techniques should be applied with majority vote, to achieve the optimal training model and enhancement technique combination.

## Conclusion

In this paper, grayscale radiograph enhancement methods aiming to achieve better classification and annotation performance is presented. Two extra color layers are augmented to simulate RGB-channeled images, as Deep Convolutional Neural Networks (dCNN) use color information for training. Due to variations in width size and height size, the radiographs are padded with cropped patches to fill up the defined size [512 x 512].

The dCNN systems Inception-v3 and Inception-ResNet-v2 were applied as image classification models. The traditional machine learning algorithm Random Forest (RF), trained with Bag-of-Keypoints visual representation, was adopted for performance comparison. Five classification schemes, each having different number of classes and categorization focus, were applied to evaluate the image enhancement techniques.

This works shows that enhancing the radiographs before training and classification, proves to obtain positive results. This is observed for the models trained with the deep learning systems Inception-v3 and Inception-ResNet-v2, as well as the traditional combination of Bag-of-Keypoints and Random Forest. For all five classification schemes, better prediction accuracies were achieved when the enhanced radiographs were used.

Prospective evaluation of annotating radiographs can be based on multi-modal image representation and hierarchical class annotation, as positive results have been presented in recent approaches.

## References

[1] Nensa F, Forsting M, Wetter A. Zukunft der Radiologie. Der Urologe. 2016;55(3):350–355. 10.1007/s00120-016-0045-1 26893136

[2] Schaer R, Müller H. A modern web interface for medical image retrieval. Swiss Medical Informatics. 2014;30.

[3] Rahman MM, Bhattacharya P, Desai BC. A Framework for Medical Image Retrieval Using Machine Learning and Statistical Similarity Matching Techniques With Relevance Feedback. IEEE Transactions on Information Technology in Biomedicine. 2007;11(1):58–69. 10.1109/TITB.2006.884364 17249404

[4] Tagare HD, Jaffe CC, Duncan JS. Synthesis of Research: Medical Image Databases: A Content-based Retrieval Approach. Journal of the American Medical Informatics Association JAMIA. 1997;4(3):184–198. 9147338 10.1136/jamia.1997.0040184 PMC61234

[5] Akgül CB, Rubin DL, Napel S, Beaulieu CF, Greenspan H, Acar B. Content-Based Image Retrieval in Radiology: Current Status and Future Directions. J Digital Imaging. 2011;24(2):208–222. 10.1007/s10278-010-9290-9 PMC3056970 20376525

[6] Roth HR, Lu L, Liu J, Yao J, Seff A, Cherry KM, et al Improving Computer-Aided Detection Using Convolutional Neural Networks and Random View Aggregation. IEEE Trans Med Imaging. 2016;35(5):1170–1181. 10.1109/TMI.2015.2482920 26441412 PMC7340334

[7] LeCun Y, Bengio Y, Hinton GE. Deep Learning. Nature. 2015;521(7553):436–444. 10.1038/nature14539 26017442

[8] Densely Connected Convolutional Networks. IEEE; 2017. Available from: https://doi.org/10.1109%2Fcvpr.2017.243.

[9] Hinton G, Deng L, Yu D, Dahl G, Mohamed Aa, Jaitly N, et al Deep Neural Networks for Acoustic Modeling in Speech Recognition: The Shared Views of Four Research Groups. IEEE Signal Processing Magazine. 2012;29(6):82–97. 10.1109/MSP.2012.2205597

[10] Abrao MS, Gonçalves MOdC, Dias JA Jr, Podgaec S, Chamie LP, Blasbalg R. Comparison between clinical examination, transvaginal sonography and magnetic resonance imaging for the diagnosis of deep endometriosis. Human Reproduction. 2007;22(12):3092–3097. 10.1093/humrep/dem187 17947378

[11] Xu Y, Mo T, Feng Q, Zhong P, Lai M, Chang EI. Deep learning of feature representation with multiple instance learning for medical image analysis. In: IEEE International Conference on Acoustics, Speech and Signal Processing, ICASSP 2014, Florence, Italy, May 4-9, 2014; 2014. p. 1626–1630. Available from: 10.1109/ICASSP.2014.6853873.

[12] Kelly L, Dungs S, Kriewel S, Hanbury A, Goeuriot L, Jones GJF, et al. Khresmoi Professional: Multilingual, Multimodal Professional Medical Search. In: Advances in Information Retrieval—36th European Conference on IR Research, ECIR 2014, Amsterdam, The Netherlands, April 13-16, 2014. Proceedings; 2014. p. 754–758. Available from: 10.1007/978-3-319-06028-6_89.

[13] Müller H, Müller W, Squire D, Marchand-Maillet S, Pun T. Performance evaluation in content-based image retrieval: overview and proposals. Pattern Recognition Letters. 2001;22(5):593–601. 10.1016/S0167-8655(00)00118-5

[14] Schaer R, Markonis D, Müller H. Architecture and applications of the Parallel Distributed Image Search Engine (ParaDISE). In: 44. Jahrestagung der Gesellschaft für Informatik, Informatik 2014, Big Data—Komplexität meistern, 22.-26. September 2014 in Stuttgart, Deutschland; 2014. p. 661–666. Available from: http://subs.emis.de/LNI/Proceedings/Proceedings232/article43.html.

[15] Lux M, Chatzichristofis SA. Lire: lucene image retrieval: an extensible java CBIR library. In: Proceedings of the 16th International Conference on Multimedia 2008, Vancouver, British Columbia, Canada, October 26-31, 2008; 2008. p. 1085–1088. Available from: http://doi.acm.org/10.1145/1459359.1459577.

[16] Lehmann TM, Güld MO, Thies C, Plodowski B, Keysers D, Ott B, et al. IRMA—Content-Based Image Retrieval in Medical Applications. In: MEDINFO 2004—Proceedings of the 11th World Congress on Medical Informatics, San Francisco, California, USA, September 7-11, 2004; 2004. p. 842–846. Available from: 10.3233/978-1-60750-949-3-842. 15360931

[17] Teare P, Fishman M, Benzaquen O, Toledano E, Elnekave E. Malignancy Detection on Mammography Using Dual Deep Convolutional Neural Networks and Genetically Discovered False Color Input Enhancement. J Digital Imaging. 2017;30(4):499–505. 10.1007/s10278-017-9993-2 PMC5537100 28656455

[18] Zuiderveld K. Graphics Gems IV. 1994; p. 474–485.

[19] Litjens GJS, Kooi T, Bejnordi BE, Setio AAA, Ciompi F, Ghafoorian M, et al A survey on deep learning in medical image analysis. Medical Image Analysis. 2017;42:60–88. 10.1016/j.media.2017.07.005 28778026

[20] Tommasi T, Caputo B, Welter P, Güld MO, Deserno TM. Overview of the CLEF 2009 Medical Image Annotation Track. In: Multilingual Information Access Evaluation II. Multimedia Experiments—10th Workshop of the Cross-Language Evaluation Forum, CLEF 2009, Corfu, Greece, September 30—October 2, 2009, Revised Selected Papers; 2009. p. 85–93. Available from: 10.1007/978-3-642-15751-6_9.

[21] Deserno TM, Ott B. 15,363 IRMA images of 193 categories for ImageCLEFmed 2009. Available from: http://dx.doi.org/10.18154/RWTH-2016-06143.

[22] Szegedy C, Vanhoucke V, Ioffe S, Shlens J, Wojna Z. Rethinking the Inception Architecture for Computer Vision. In: 2016 IEEE Conference on Computer Vision and Pattern Recognition, CVPR 2016, Las Vegas, NV, USA, June 27-30, 2016; 2016. p. 2818–2826. Available from: 10.1109/CVPR.2016.308.

[23] Szegedy C, Ioffe S, Vanhoucke V, Alemi AA. Inception-v4, Inception-ResNet and the Impact of Residual Connections on Learning. In: Proceedings of the Thirty-First AAAI Conference on Artificial Intelligence, February 4-9, 2017, San Francisco, California, USA.; 2017. p. 4278–4284. Available from: http://aaai.org/ocs/index.php/AAAI/AAAI17/paper/view/14806.

[24] Krizhevsky A, Sutskever I, Hinton GE. ImageNet Classification with Deep Convolutional Neural Networks. In: Proceedings of the 25th International Conference on Neural Information Processing Systems—Volume 1. NIPS’12. USA: Curran Associates Inc.; 2012. p. 1097–1105. Available from: http://dl.acm.org/citation.cfm?id=2999134.2999257.

[25] Goodfellow I, Bengio Y, Courville A. Deep Learning Adaptive computation and machine learning series. The MIT Press; 2016.

[26] Pizer SM, Amburn EP, Austin JD, Cromartie R, Geselowitz A, Greer T, et al Adaptive Histogram Equalization and Its Variations. Comput Vision Graph Image Process. 1987;39(3):355–368. 10.1016/S0734-189X(87)80186-X

[27] Buades A, Coll B, Morel JM. A Non-Local Algorithm for Image Denoising. In: Proceedings of the 2005 IEEE Computer Society Conference on Computer Vision and Pattern Recognition (CVPR’05)—Volume 2—Volume 02. CVPR’05. Washington, DC, USA: IEEE Computer Society; 2005. p. 60–65. Available from: 10.1109/CVPR.2005.38.

[28] Abadi M, Agarwal A, Barham P, Brevdo E, Chen Z, Citro C, et al. TensorFlow: Large-Scale Machine Learning on Heterogeneous Systems; 2015. Available from: https://www.tensorflow.org/.

[29] Russakovsky O, Deng J, Su H, Krause J, Satheesh S, Ma S, et al ImageNet Large Scale Visual Recognition Challenge. International Journal of Computer Vision (IJCV). 2015;115(3):211–252. 10.1007/s11263-015-0816-y

[30] He K, Zhang X, Ren S, Sun J. Deep Residual Learning for Image Recognition. In: Conference on Computer Vision and Pattern Recognition CVPR. IEEE Computer Society; 2016. p. 770–778.

[31] He K, Zhang X, Ren S, Sun J. Identity Mappings in Deep Residual Networks. In: European Conference on Computer Vision ECCV. vol. 9908 of Lecture Notes in Computer Science. Springer; 2016. p. 630–645.

[32] Breiman L. Random Forests. Machine Learning. 2001;45(1):5–32. 10.1023/A:1010933404324

[33] Csurka G, Dance CR, Fan L, Willamowski J, Bray C. Visual categorization with bags of keypoints. In: In Workshop on Statistical Learning in Computer Vision, ECCV; 2004. p. 1–22.

[34] Lazebnik S, Schmid C, Ponce J. Beyond Bags of Features: Spatial Pyramid Matching for Recognizing Natural Scene Categories. In: Proceedings of the 2006 IEEE Computer Society Conference on Computer Vision and Pattern Recognition—Volume 2. CVPR’06; 2006. p. 2169–2178.

[35] Zhang H, Berg AC, Maire M, Malik J. SVM-KNN: Discriminative Nearest Neighbor Classification for Visual Category Recognition. In: Proceedings of the 2006 IEEE Computer Society Conference on Computer Vision and Pattern Recognition—Volume 2. CVPR’06; 2006. p. 2126–2136.

[36] Vedaldi A, Fulkerson B. VLFEAT: an open and portable library of computer vision algorithms. In: Proceedings of the 18th International Conference on Multimedia 2010, Firenze, Italy, October 25-29, 2010; 2010. p. 1469–1472. Available from: http://doi.acm.org/10.1145/1873951.1874249.

[37] Li FF, Perona P. A Bayesian Hierarchical Model for Learning Natural Scene Categories. In: Proceedings of the 2005 IEEE Computer Society Conference on Computer Vision and Pattern Recognition (CVPR’05)—Volume 2—Volume 02. CVPR’05; 2005. p. 524–531.

[38] Indyk P, Motwani R. Approximate Nearest Neighbors: Towards Removing the Curse of Dimensionality. In: Proceedings of the 30th Annual ACM Symposium on Theory of Computing. STOC’98. New York, NY, USA: ACM; 1998. p. 604–613.

[39] Hartigan JA, Wong MA. A k-means clustering algorithm. JSTOR: Applied Statistics. 1979;28(1):100–108.

[40] Wolpert DH, Macready WG. No Free Lunch Theorems for Optimization. Transactions on Evolutionary Computing. 1997;1(1):67–82. 10.1109/4235.585893

